# Genome-wide SNP analysis of Japanese Thoroughbred racehorses

**DOI:** 10.1371/journal.pone.0218407

**Published:** 2019-07-24

**Authors:** Jeffrey A. Fawcett, Fumio Sato, Takahiro Sakamoto, Watal M. Iwasaki, Teruaki Tozaki, Hideki Innan

**Affiliations:** 1 SOKENDAI (The Graduate University for Advanced Studies), Hayama, Kanagawa 240-0193, Japan; 2 RIKEN iTHEMS, Wako, Saitama 351-0198, Japan; 3 Hidaka Training and Research Center, Japan Racing Association, Hokkaido 057-0171, Japan; 4 Genetic Analysis Department, Laboratory of Racing Chemistry, Utsunomiya, Tochigi 320-0851, Japan; Kansas State University, UNITED STATES

## Abstract

The domestication process of plants and animals typically involves intense inbreeding and directional selection for various traits. Here, we genotyped 370 Japanese Thoroughbred horses using the recently developed 670k SNP array and performed various genome-wide analysis also using genotype data of other horse breeds. We identified a number of regions showing interesting patterns of polymorphisms. For instance, the region containing the *MC1R* locus associated with chestnut coat color may have been targeted by selection for a different mutation much earlier on than the recent selection for chestnut color. We also identified regions that show signatures of selection specific to Thoroughbreds. In addition, we found that intense inbreeding early in the history of the Thoroughbred breed and also before the formation of the breed has a significant impact on the genomic architecture of modern Thoroughbreds. Our study demonstrates that the horse 670k array can be utilized to gain important insight into the domestication process of horses and to understand the genetic basis of the phenotypic diversity in horses.

## Introduction

Humans have been selectively breeding several plants and animals for a wide range of desirable traits throughout history. This domestication process offers an excellent opportunity to understand the genetic basis of phenotypic variation, and also to understand how forces such as directional selection, population bottleneck, and inbreeding shape the genome [1, 2]. A number of recent studies using ancient genomes have provided important insights into the domestication history of horses [3–7]. It seems that the domestication occurred around 5,500 years ago, a process which likely involved a population bottleneck and reduction in genetic variation. Since then, several breeds that exhibit high phenotypic variation have been established. One of the most popular breed is the Thoroughbred, which originated around the 17th century from a small number of stallions and mare, and have since been selectively bred for traits such as speed and stamina [8–10].

Despite the economical value of Thoroughbreds, genetic information has traditionally not been considered in the breeding of Thoroughbreds. Even with the progress in DNA sequencing in the recent years and the availability of a reference genome [11], genomic information has hardly been utilized. Only recently, a SNP in the myostatin locus that affects the optimal racing distance (e.g. speed-type suited for short distance races vs stamina-type suited for long distance races) was identified [12–14]. This SNP became the first molecular marker to be developed in the breeding of Thoroughbreds. Yet, Thoroughbred horses are an interesting system for genetic studies because detailed pedigree information is available for most horses, various phenotypes are well characterized, and they have been inbred and subjected to strong directional selection for several generations in different parts of the world [15–18]. Moreover, the recent development of the 670k SNP array [19] provides a cost-effective way to obtain genome-wide SNP data.

Here, we investigated the genetic variation of Japanese Thoroughbred racehorses by genotyping 370 Japanese Thoroughbreds using the 670k SNP array. Our study provides insight into the impact of inbreeding and artificial selection on the genetic makeup of Thoroughbred horses. Our study also provides a platform to identify genomic loci associated with various traits which should help improve the breeding of Thoroughbreds.

## Results

### Overview

We surveyed the genotypes of a total of 370 Japanese Thoroughbred racehorses using the Axiom Equine Genotyping Array [19]. These horses consist of 198 males and 172 females. Most horses (331 out of 370) have at least one half or full sibling also included in this dataset, although there are no dominant sires as artificial insemination is not allowed for Thoroughbred horses. The most common sire in our sample is shared by 31 half-sibs and the sires are not present in our sample. The complete pedigree information for at least the past 5 generations, and in most cases more, was available for all horses. Out of the 670,796 sites surveyed, 352,974 autosomal SNPs were genotyped in >99% of the samples with a minor allele frequency (MAF) of ≥1%, and 305,297 with MAF of ≥5%. Thus, approximately half of the sites on the Axiom 670K array were informative for surveying the genetic variation in Japanese Thoroughbreds. As our samples contained several half-sib families, we used *hsphase* [20, 21], a program specifically designed to phase half-sib families, to phase each half-sib family containing 10 or more half-sibs (117 samples in total). The remaining samples were phased using BEAGLE [22]. The linkage disequilibrium (LD) between the SNPs within 1 Mb was calculated using the phased haplotypes and the decay of LD against the physical distance was examined [23]. The shape of the decay of LD was similar to what was reported in [19], although the *r*^2^ in our study is slightly lower and drops below 0.2 at ∼70kb (S1 Fig), which may be because we calculated LD based on the phased haplotypes rather using the *r*^2^ between the genotypes.

**Fig 1:**
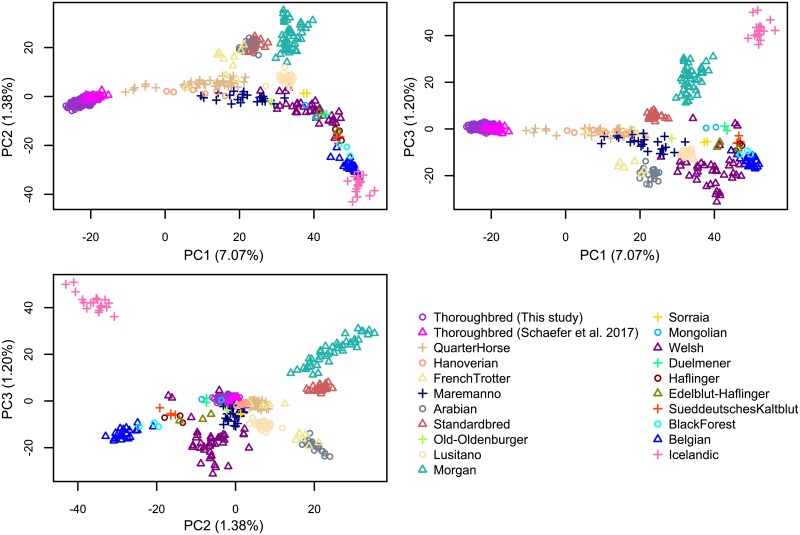
Results of principal component analysis (PCA). SNPs from the 370 Thoroughbreds genotyped in this study and the 311 samples from 20 breeds of [19] were used. The result using a subset of the Japanese Thoroughbreds is in S2 Fig.

**Fig 2:**
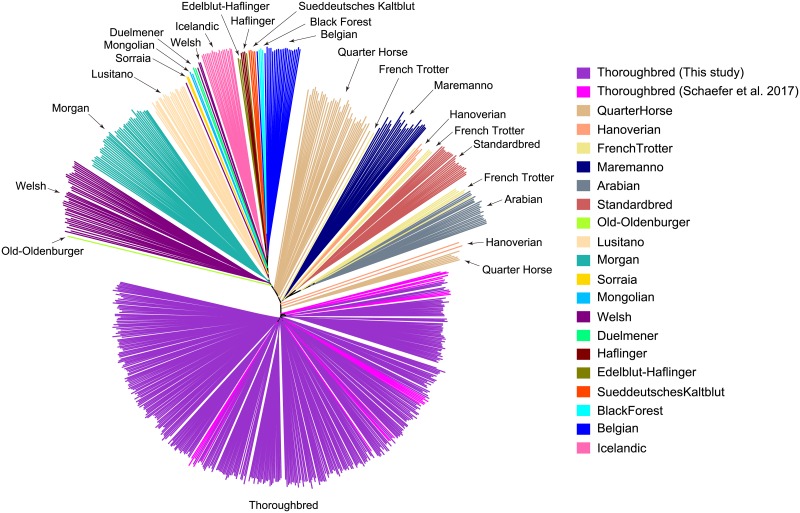
Unrooted Neighbor-Joining tree. SNPs from the 370 Thoroughbreds genotyped in this study and the 311 samples from 20 breeds of [19] were used. A tree with bootstrap values for the major branches is shown in S3 Fig.

**Fig 3:**
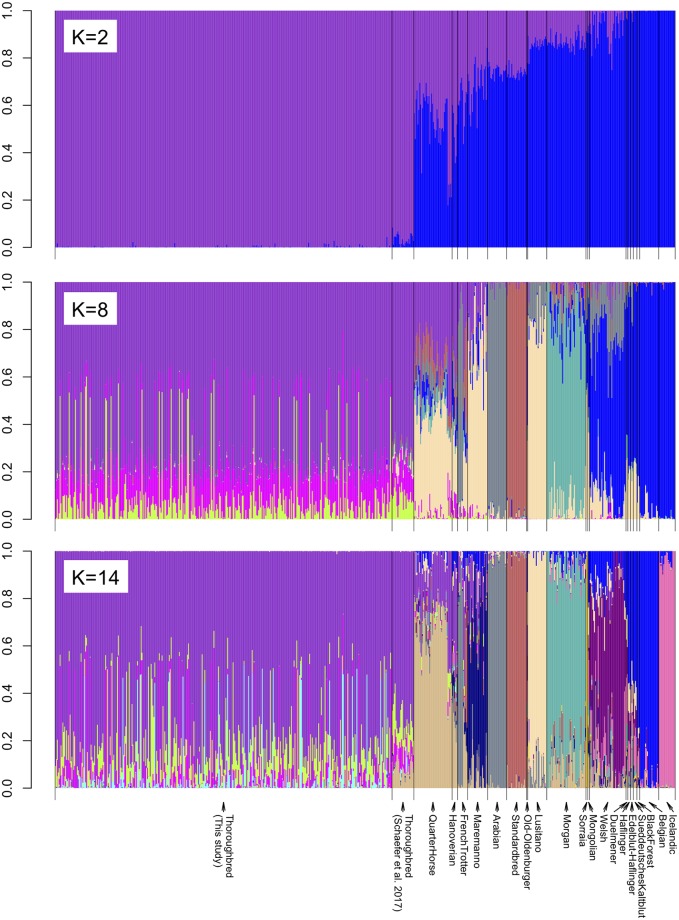
Results of STRUCTURE analysis of *K* = 2, 8, 14 SNPs from the 370 Thoroughbreds genotyped in this study and the 311 samples from 20 breeds of [19] were used. The result using a subset of the Japanese Thoroughbreds is in S4 Fig.

**Fig 4:**
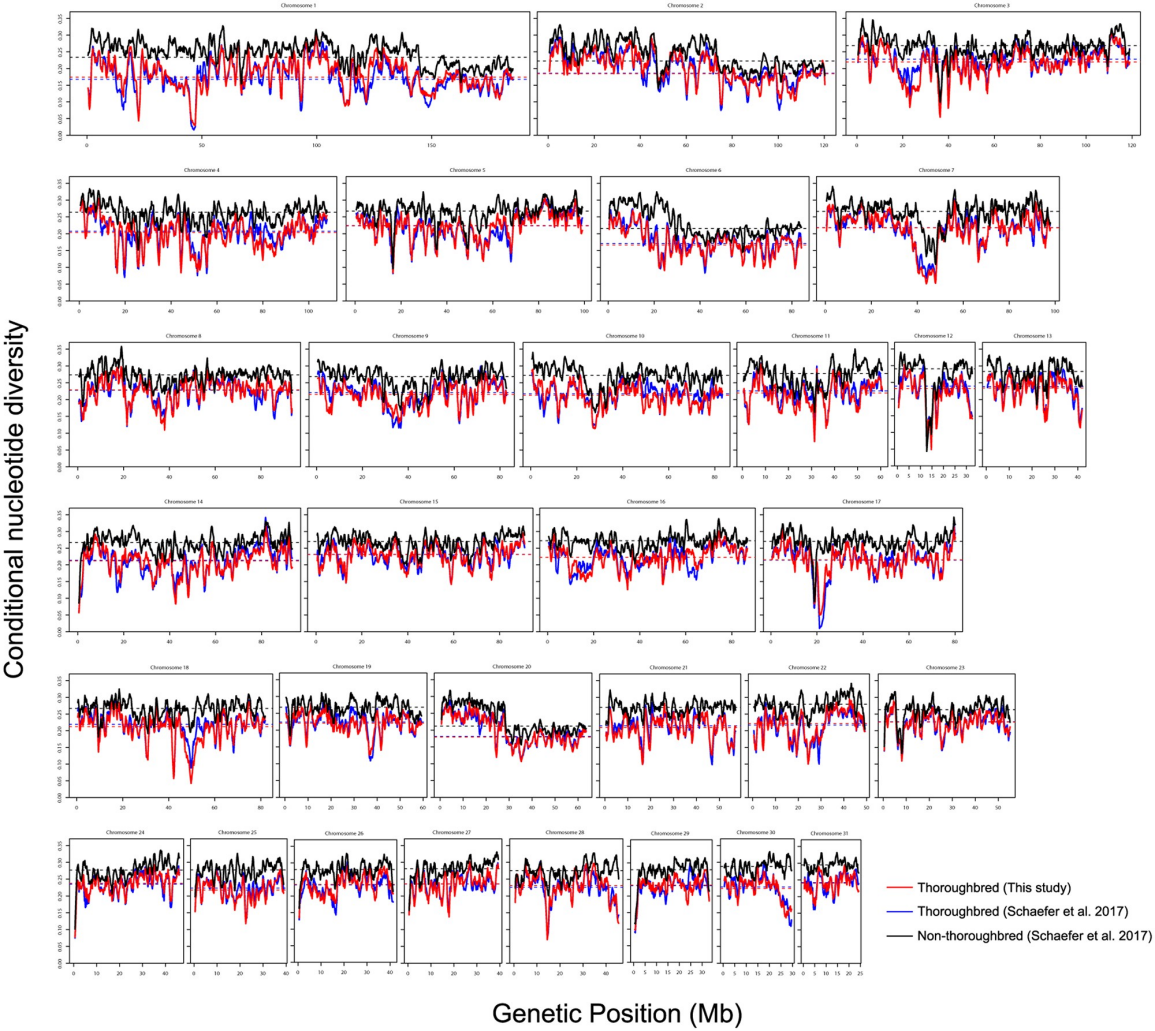
Genome-wide distribution of conditional nucleotide diversity. The distributions based on the SNPs from the 370 Thoroughbreds of this study, 24 Thoroughbreds of [19], and 287 non-Thoroughbreds of [19] are shown in red, blue, and black, respectively. The distributions are based on values calculated for each sliding window of 1 Mb with an increment of 200 kb. The average values for each chromosome are shown by horizontal dotted lines.

We then compared the genetic structure of our samples with those from a recently published study which includes several other horse breeds and were genotyped using the same 670K SNP array [19]. We performed principal component analysis (PCA) [24, 25], constructed Neighbor-Joining (NJ) trees [26], and STRUCTURE analysis [27] using the genotypes of 370 samples from this study and 311 samples of 20 different breeds from the study of [19]. As expected, the results of PCA, NJ tree, and STRUCTURE all clearly show that our samples form a distinct cluster together with the other Thoroughbreds from [19] (Figs 1, 2 and 3, S2, S3 and S4 Figs). The relationship between our samples and the other breeds are largely consistent with previous studies [28, 29]. It is known that Thoroughbreds have been crossed to other breeds such as the Quarter Horse, Hanoverian, Marremano, and the French Trotter. Indeed, signatures of cross breeding between Thoroughbreds and these breeds can be observed in the results of STRUCTURE (Fig 3). The PCA and STRUCTURE results show that a number of individuals of the Quarter Horse and the Hanoverian breed are especially closely related to Thoroughbreds. The results of STRUCTURE at *K* = 14 indicates a slightly stronger degree of admixture between the Quarter Horse and the Thoroughbreds of Schaefer *et al*. [19] compared to the Thoroughbreds of this study. This may be due to the large amount of gene flow from the American Thoroughbred horses to the Quarter Horses, as the samples in this study are all Japanese Thoroughbreds. As the much larger sample size of the Japanese Thoroughbreds compared to the other breeds might affect the PCA and STRUCTURE results, we performed the same analyses using a subset of the Japanese Thoroughbreds (S2 and S4 Figs). The results are generally consistent, although the relationship between the Japanese Thoroughbreds and the Thoroughbreds of Schaefer *et al*. [19] do depend on which Japanese Thoroughbreds are selected as a subset. We also examined the genome-wide distribution of the level of conditional nucleotide diversity, i.e., nucleotide diversity conditional on the genotype SNPs in the Axiom Equine Genotyping Array. We plotted the distribution separately for the Thoroughbreds used in this study, the Thoroughbreds from [19], and the non-Thoroughbred breeds from [19] (Fig 4). The distribution of the Thoroughbreds of this study and those from [19] are highly similar, although there are a few regions which differ. The average diversity level is much lower in Thoroughbreds as the distribution of non-Thoroughbreds contains many different breeds. The diversity level in Thoroughbreds is markedly reduced compared to the other breeds in several regions, possibly due to artificial selection specific to Thoroughbreds (see below for details).

### Reconstructing haplotypes of sires

**Fig 5:**
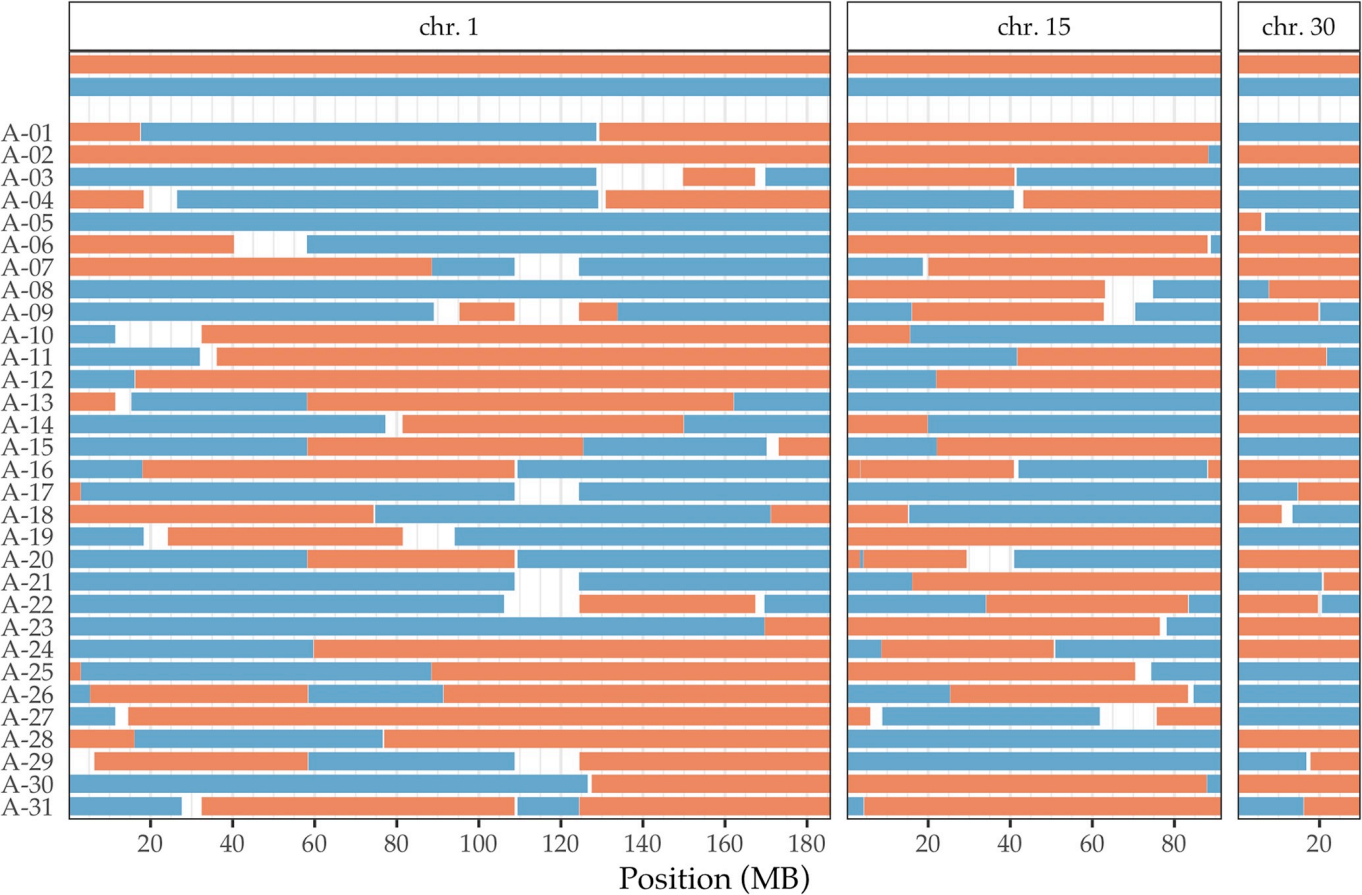
Haplotype block structures of the chromosomes inherited from the sire in a half-sib family inferred by *hsphase*. The two homologous chromosomes of the sire are shown at the top in red and blue, and the chromosomes inherited from the sire in each genotyped half-sib (A-01 to A-31) are shown below. Examples of chromosomes 1, 15, and 30 of the largest half-sib family (Sire A) are shown. Plots containing all chromosomes of all half-sib families are shown in S5 Fig.

**Fig 6:**
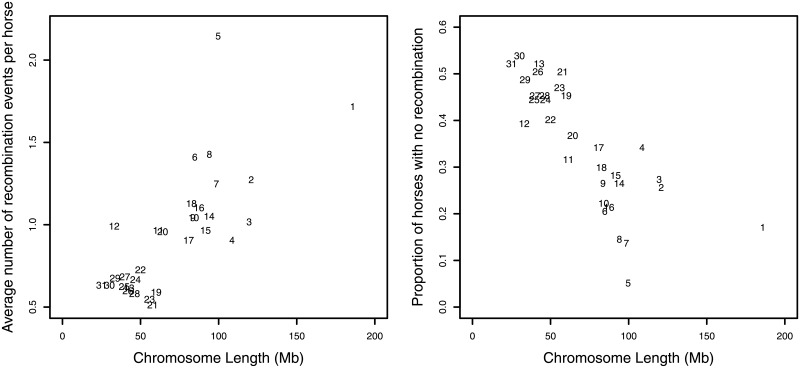
Estimated amount of recombination events in half-sib families. Average number of inferred recombination events per horse (left panel) and the proportion of horses with no inferred recombination events (right panel) for each chromosome plotted against the length of the chromosome.

The presence of several half-sibs sharing the same sire makes it possible to accurately phase the chromosomes of each half-sib, and also infer the phased chromosomes of the sire even though the sires were not genotyped. This allows us to examine how the homologous chromosomes of the sire are recombined and passed on to each offspring. Based on the results of *hsphase* for the half-sib families, we were able to infer the haplotype blocks in the half-sibs inherited from the sires and the recombination events that occurred during meiosis. Examples of three chromosomes of the largest half-sib family are shown in Fig 5, whereas the results of all chromosomes of all the half-sib families with 10 or more half-sibs are shown in S5 Fig. The two homologous chromosomes of the sire (red and blue) are shown at the top together with the recombinant chromosomes of the half-sibs that were inherited from the sire. Although inaccurate phasing tends to result in an unrealistic number of recombination events inferred, the inferred number of recombination events on average per chromosome was ∼1, which is similar to what was previously reported for horses [30]. The number of recombination events were similar for both small and large half-sib families (Table 1). Thus, the phased haplotypes and the inferred chromosomes of the sires should be relatively accurate, even if there may be some errors. As expected, the number of recombinations was strongly correlated to the size of chromosomes (Fig 6). Although it is thought that the formation of at least one chiasma per chromosome during meiosis is crucial [31], the daughter cells receive either the recombinant or the non-recombinant chromatid. As such, especially for small chromosomes where only one chiasma is expected to occur, some offspring inherit chromosomes with no recombination events [32]. Indeed, we found that the proportion of horses without recombination events decreased in proportion to the chromosome length as expected, with approximately a half of the horses showing no recombinations in the short chromosomes (Fig 6).

**Table 1:** Average number of inferred recombination events per horse for each half-sib family.

Sire ID	# of half-sibs	# of Recombinations
A	31	31.71
B	25	29.48
C	19	25.37
D	17	28.59
E	15	28.73
F	10	31.90

### Inbreeding

Thoroughbreds are highly inbred with a closed breeding system where foals with a non-Thoroughbred sire or dam cannot be registered as Thoroughbreds. Very strong inbreeding (e.g. between first cousins) is generally avoided at least in recent years. All modern Thoroughbreds can be traced back to a small number of founder stallions and mares, indicating severe bottleneck during the establishment of the breed [8–10, 18]. The domestication of horses is also thought to have involved a severe bottleneck process [4, 5]. Our data should allow us to examine how inbreeding has affected the genomes of modern Thoroughbreds. Moreover, because the pedigree information is available for all our samples, we can examine the difference between the inbreeding coefficient calculated based on pedigree and based on genomic data.

**Fig 7:**
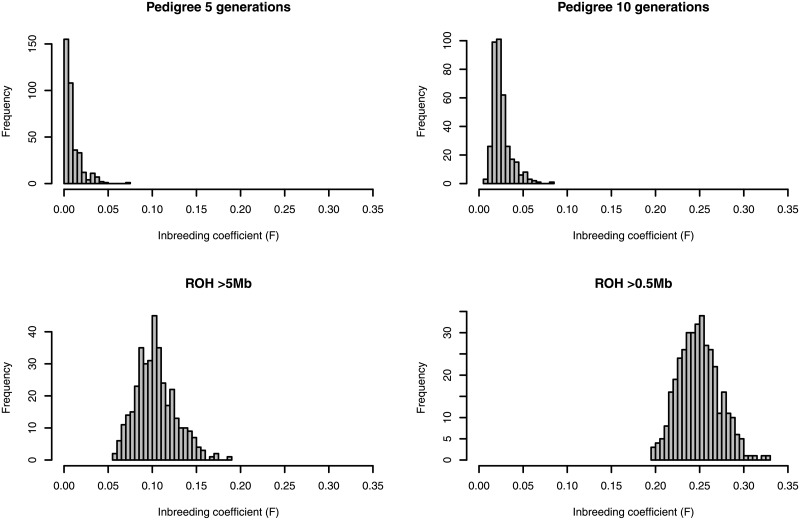
Histogram of inbreeding coefficients based on pedigree and based on runs of homozygosity. Inbreeding coefficients were calculated for each of the 370 Thoroughbreds.

**Fig 8:**
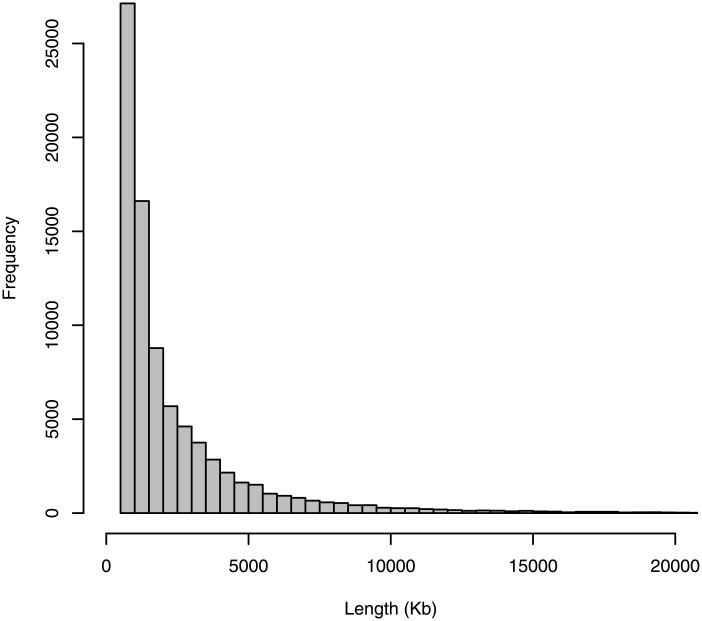
Histogram of the distribution of the lengths of ROH segments.

We first calculated the inbreeding coefficient *F* for each individual based on the pedigree of the past 5 (*F*_*PED*5_) and 10 (*F*_*PED*10_) generations (Fig 7). All horses had complete pedigree records for the past 5 generations whereas all horses had some missing records in their 10 generation pedigree. As expected, the inbreeding coefficient based on the 5 generation pedigree (*F*_*PED*5_) was low for most individuals, with most horses having *F*_*PED*5_ <0.03. The inbreeding coefficients were much larger when calculated based on pedigrees of the past 10 generations (*F*_*PED*10_), even though these estimates should be underestimates due to the incompleteness of the pedigree record. We then calculated the inbreeding coefficient *F* from the genome-wide SNP data based on runs of homozygosity (ROH) of >0.5 Mb (*F*_*ROH*>0.5*Mb*_) and >5 Mb (*F*_*ROH*>5*Mb*_) (Fig 7). ROH is thought to be the most appropriate way to measure the degree of inbreeding and is becoming more and more popular in recent years [33–35]. ROH with a large threshold (e.g. >5 Mb) should detect more recent inbreeding, whereas ROH with smaller thresholds (e.g. >0.5 Mb) should allow the detection of much older inbreeding, e.g. from ∼50-100 generations ago [33], at the expense of detecting more segments that are allozygous rather than autozygous. Considering that Thoroughbreds are ∼25-30 generations old [36], *F*_*ROH*>0.5*Mb*_ should also be capturing inbreeding that occurred long before the Thoroughbred breed was established. By contrast, *F*_*ROH*>5*Mb*_ should mostly be capturing inbreeding that occurred within Thoroughbreds, but not restricted to the past 10 generations. The inbreeding coefficients estimated based on ROH were considerably larger than those based on pedigree (Fig 7). Each horse contained on average 224 ROH segments of >0.5 Mb (Table 2). The average length of each ROH segment was 2.49 Mb, although the highest proportion of ROH segments were 0.5-1 Mb (Fig 8). The amount of the genome covered by ROH segments of >0.5 Mb ranged from 438 Mb to 736 Mb, with a mean of 551 Mb, which account for 24.5% of the genome. ROH segments of >5 Mb, which reflect more recent inbreeding, account for an average of 233 Mb, or 10.4% of the genome. *F*_*ROH*_ and *F*_*PED*_ in our samples were positively correlated. The correlation between *F*_*ROH*>0.5*Mb*_ and *F*_*PED*5_, *F*_*PED*10_ was 0.35, 0.37, respectively, whereas the correlation between *F*_*ROH*>5*Mb*_ and *F*_*PED*5_, *F*_*PED*10_ was 0.34, 0.37, respectively. These values are much lower than those reported in other studies, which are mostly on cattle and typically ∼0.5-0.7 [34].

**Table 2:** Mean, minimum, and maximum genome length (kilobases) covered by ROH and mean, minimum, and maximum number of ROH segments per individual.

	Mean *L*_*ROH*_	Stdev	Min	Max	Mean *n*_*ROH*_	Stdev	Min	Max
*F*_*ROH*>0.5*Mb*_	550974	51512	437768	736408	224.1	12.8	191	268
*F*_*ROH*>5*Mb*_	232745	49932	124629	415195	26.2	4.8	15	43

### Association studies

**Fig 9:**
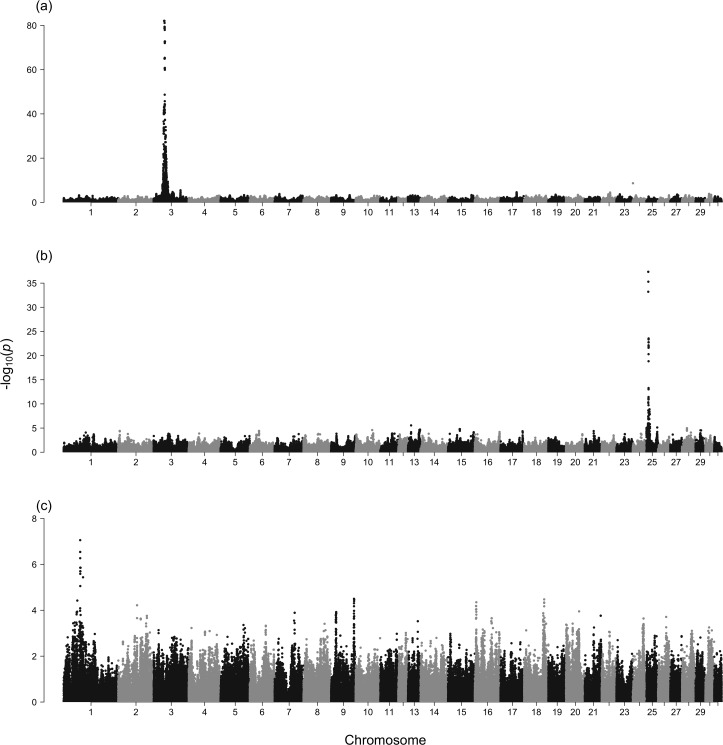
Genome-wide association analysis for known color coat loci and racing performance. Manhattan plot for (a) Chestnut coat color, (b) gray coat color, and (c) racing performance.

Population genetic analysis using genome-wide SNP data can be very powerful in identifying loci responsible for various phenotypes. In particular, genome-wide association studies (GWAS) is often used to identify candidate regions associated with traits of interest. To examine the utility of our dataset for GWAS, we first performed GWAS for 2 known color coat loci, gray and chestnut. It is known that all Thoroughbred horses (excluding very rare white horses) with a mutation in the *Gray* locus (*STX17*) on chromosome 25 have a gray coat color [37], and of the non-gray horses, all horses homozoygous for a mutation in the *Extension* locus (*MC1R*) on chromosome 3 have a chestnut coat color [38]. Our samples consisted of 18 gray horses, 94 chestnut horses, and 258 with other coat colors. Although it is common practice to remove related individuals as much as possible when performing GWAS, this is not practical with Thoroughbreds where many individuals are related to each other. We therefore used a mixed model approach implemented in GEMMA [39], which first estimates the degree of relatedness between each individual based on the SNP data, and then controls for the relatedness using a linear mixed model. A region including the *Gray* locus on chromosome 25 showed an extremely strong association with gray coat color, whereas a large region on chromosome 3 including the *Extension* locus showed an extremely strong association with the chestnut phenotype (Fig 9). Although a study using the previously designed 50k array also detected the strong association with the chestnut coat color, a significant association with the gray coat color was not detected [28]. This illustrates the utility of the high density 670k array in association studies. We next attempted to identify loci that are associated with racing performance. We performed GWAS between 29 high-performance horses with lifetime earnings of ≥30 million JPY and 141 low-performance horses with lifetime earnings of <5 million JPY despite having raced at least 3 times. Although racing ability is a complex trait and the lifetime earnings can be influenced by many different factors, some regions, in particular one on chromosome 1 showed a strong association (Fig 9). This region on chromosome 1 contains *MYPN*, a gene encoding for myopalladin, which is expressed in skeletal and cardiac muscles and has an important role in regulating actin organization in humans [40]. One previous study reported this gene as undergoing positive selection in horses [3]. Another recent study found that this region is significantly associated with differences in height in American Belgian Draft Horses [41].

### Targets of artificial selection

**Fig 10:**
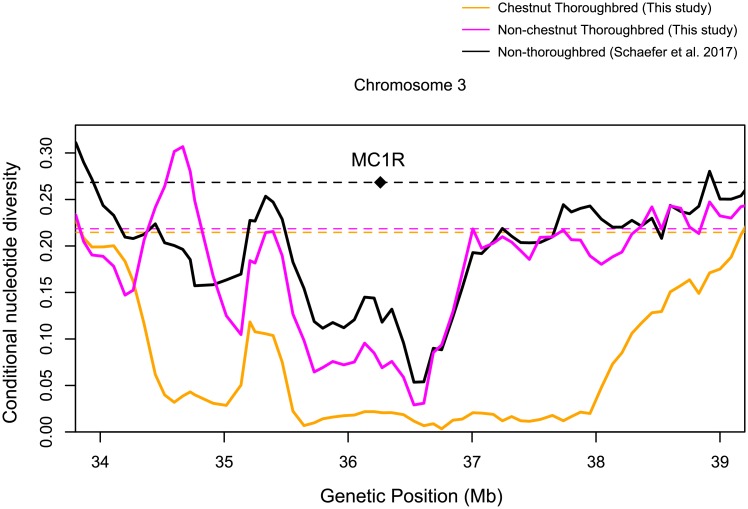
Putative selective sweep around the *MC1R* locus. The distributions based on the SNPs from the 94 Thoroughbreds with chestnut coat color, 276 Thoroughbreds with non-chestnut coat color of this study, and 287 non-Thoroughbreds of [19] are shown in orange, magenta, and black, respectively. The distributions are based on values calculated for each sliding window of 400 kb with an increment of 80 kb. The average values for each chromosome are shown by horizontal dotted lines.

Another powerful approach to identify loci of interest in domesticated species is to search for targets of selection. Horses have been subject to artificial selection during the process of domestication and Thoroughbreds are currently subject to strong directional selection [5]. Genomic regions targeted by such artificial selection should typically show reduced levels of nucleotide diversity. By looking at the genome-wide distribution of conditional nucleotide diversity (Fig 4), we can see that a number of regions show reduced levels of diversity, including regions that have been previously reported. For instance, the region on chromosome 3 containing the *MC1R* gene (36,259,305-36,260,258) responsible for the chestnut coat color showed reduced levels of diversity in Thoroughbreds and in the other breeds (Fig 10), consistent with previous studies [4, 14]. It has also been demonstrated by analyzing archaeological samples that the chestnut allele in *MC1R* rapidly increased from around the Bronze age 3,000-4,000 years ago most probably due to selective breeding [42]. However, we found our results puzzling because the proportion of chestnut horses in our samples of Japanese Thoroughbreds was only ∼25% (94/370). We therefore examined the distribution of polymorphisms separately for the chestnut and non-chestnut Thoroughbreds. As expected, the chestnut samples showed very low levels of polymorphisms over a large region containing *MC1R*, consistent with strong recent selection for the chestnut coat color. Interestingly, the non-chestnut horses also show reduced levels of diversity, although the reduction is much more localized (Fig 10). This pattern cannot be explained by selective breeding of chestnut horses, and instead may reflect a selective sweep targeting a different loci that occurred much earlier in the evolution of horses. One gene in this region that may be of interest is a gene encoding for a putative olfactory receptor 7A, as a recent study reported an enrichment for olfactory receptor genes in genes showing signatures of selection [3].

**Fig 11:**
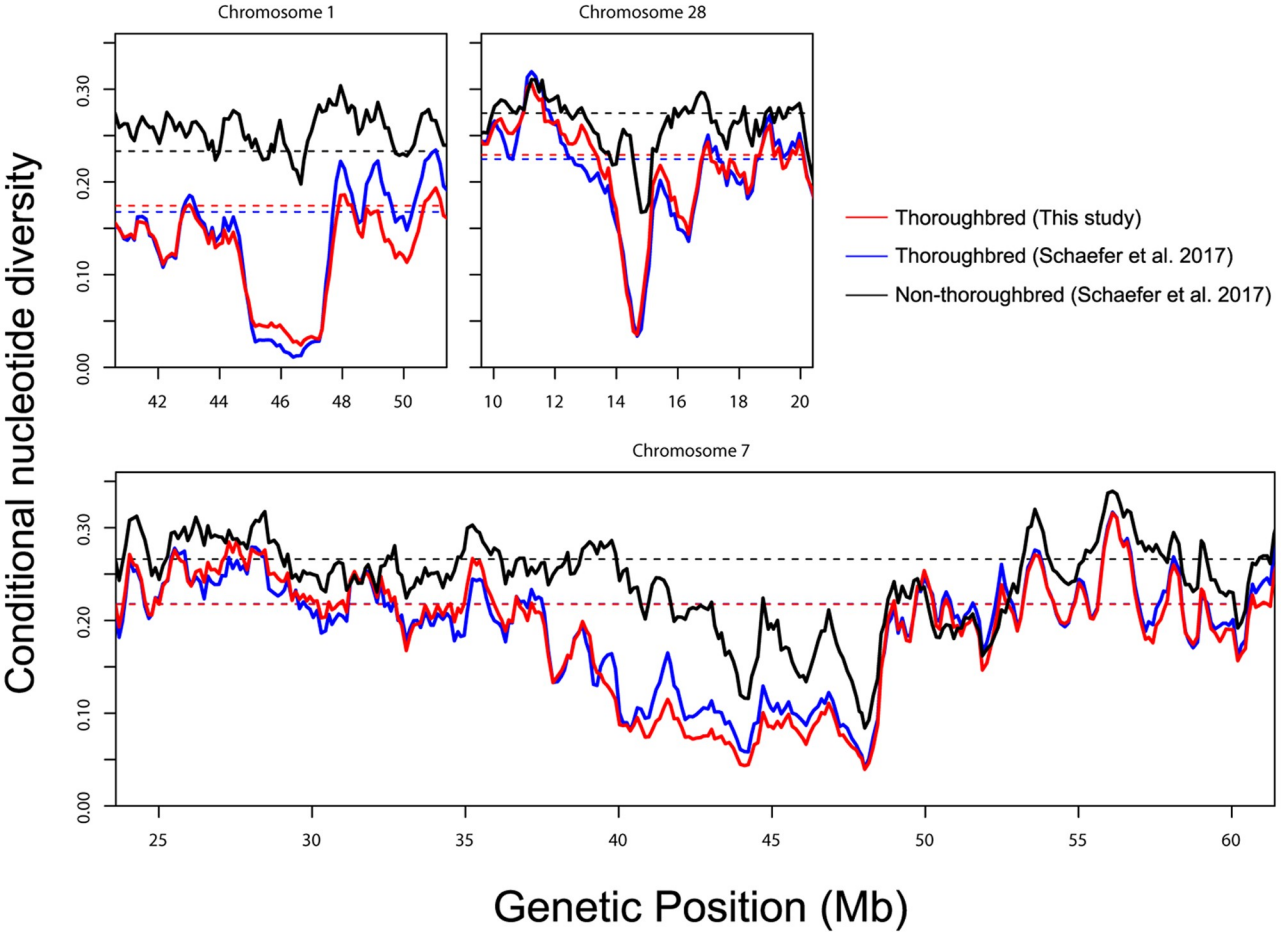
Examples of regions showing signatures of selective sweeps. The distributions based on the SNPs from the 370 Thoroughbreds of this study, 24 Thoroughbreds of [19], and 287 non-Thoroughbreds of [19] are shown in red, blue, and black, respectively. The distributions are based on values calculated for each sliding window of 600 kb with an increment of 120 kb. The average values for each chromosome are shown by horizontal dotted lines.

A number of other regions showed reduced levels of polymorphisms, such as a large region on chromosome 7 that was also reported by [4] as a target of selection due to a high log-ratio of the Watterson estimator ${\hat{\theta }}_{w}$ between the pre-domesticated and domestic horse genomes and a decrease in Tajima’s *D*. In addition, we were able to identify a number of regions where Thoroughbreds show a much more pronounced reduction in diversity than the other breeds (Fig 11). For instance, one region on chromosome 1 shows a drastic reduction of diversity in Thoroughbreds whereas reduced diversity is not observed in the other breeds. Protein-coding genes present in this region are *PCDH15*, a gene encoding protocadherin 15 which is necessary for hearing and balance functions [43], and *ZWINT*, a gene involved in kinetochore function [44]. Another region on chromosome 28 containing the *KITLG* gene that is associated with fertility, neural cell development and haematopoiesis has been previously reported as showing signatures of selection [3, 4, 45]. Interestingly, we found that this region indeed shows reduced levels of diversity in Thoroughbreds but not so much in the other breeds.

**Fig 12:**
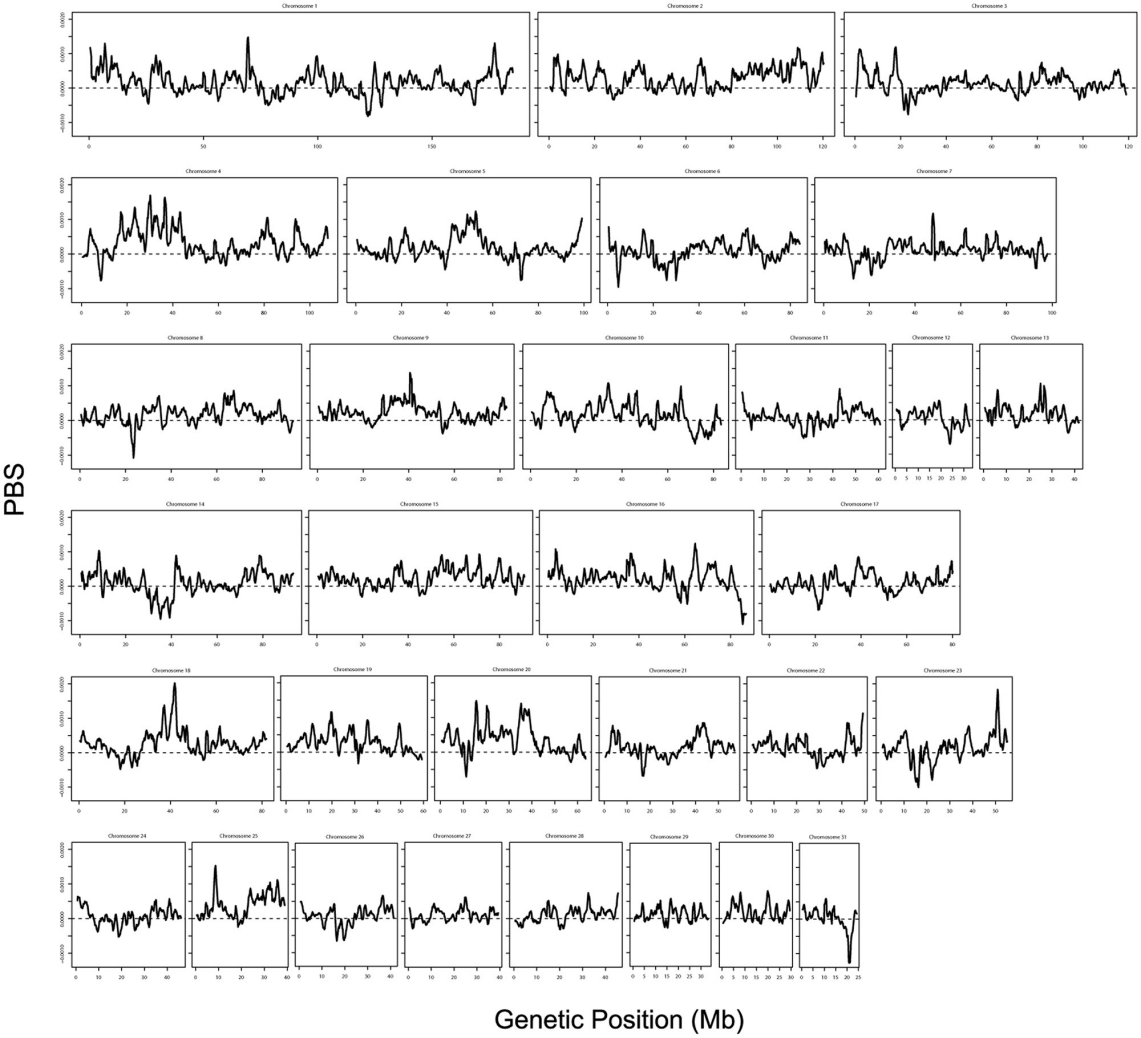
Genome-wide distribution of population branch statistic (PBS) values. The PBS values representing the branch length of Japanese Thoroughbreds since their divergence from the other Thoroughbreds and the non-Thoroughbred horses were calculated based on the SNPs from the 370 Thoroughbreds of this study, 24 Thoroughbreds of [19], and 287 non-Thoroughbreds of [19]. The distribution is based on the PBS values calculated for each sliding window of 1 Mb with an increment of 200 kb.

We also searched for targets of selection specific to Japanese Thoroughbreds by estimating changes in allele frequency specific to Japanese Thoroughbreds. The population branch statistic (PBS) [46] was calculated based on the pairwise *F*_*ST*_ values between the Japanese Thoroughbreds, Thoroughbreds of [19], and all the other horse breeds over each genomic region. The genome-wide distribution reveals a number of regions with high PBS values which could represent targets of selection specific to Japanese Thoroughbreds (Fig 12). The top 10 regions with highest PBS values are provided in S1 Table with the protein-coding genes in each region and the Gene Ontology terms for each gene. The region with the highest values of PBS contains *CHL1*, a gene encoding for a cell adhesion molecule L1-like protein. CHL1 deficient mice shows impaired social behavior, altered behavior in novel environments, and deficits in information processing [47, 48]. Polymorphism in the human homolog is associated with schizophrenia [49]. Other genes related to social behavior and schizophrenia have been reported as targets of selection in horses, possibly due to their role in learning capabilities and social interactions that are crucial to the success of various breeds of domesticated horses [4, 5].

## Discussion

Population genomic studies have proven extremely useful in identifying genomic loci associated with agronomically important traits and also to understand the domestication process of these species [1, 2]. Here, we genotyped 370 Japanese Thoroughbreds and analyzed their genetic structure. We used this data to identify interesting genomic regions such as regions associated with various phenotypic traits and regions that were likely targeted by artificial selection. In particular, a number of regions showed reduced conditional nucleotide diversity in Thoroughbreds compared to other breeds, suggesting that these regions have been recent targets of selection in Thoroughbreds. It is important to note that population genetic analyses such as selection scans using SNP arrays may suffer from biases in the array design. Nevertheless, the regions we detected largely overlap with those detected by studies based on resequencing of the whole genome. Thus, even if the results may be slightly less reliable than those based on resequencing, our results demonstrate that the high density 670k Equine SNP array can be used as an effective approach to search for targets of selection in horses and generate testable hypotheses. For instance, while the frequency of the chestnut allele most likely increased due to recent positive selection, our results suggest that a neighboring loci of the *MC1R* gene may have been the target of a selective sweep much earlier on [4, 14, 42], prior to the selective breeding of chestnut horses. Further detailed analysis of this region may provide important insights into the domestication history of horses. We also identified a number of regions showing reduced levels of polymorphisms in Thoroughbreds and changes in allele frequency specific to Japanese Thoroughbreds, which may contain polymorphisms associated with traits that characterize this breed.

Our results offer interesting insights regarding the effect of historical inbreeding in Thoroughbreds. We found that the degree of inbreeding estimated based on pedigree information considerably underestimates the degree of inbreeding observed in the genome as measured by segments of ROH. This is likely due to the excess of historical inbreeding that is not captured by the recent pedigree. One recent study examined the pedigree-based inbreeding coefficients in 135,572 Australian Thoroughbreds by compiling an extensive pedigree dating back to the founders of the population [18]. They found that most of the inbreeding observed in the current population is accounted for by a small number of ancestors early in the formation of the breed. Our results are consistent with the impact of these early Thoroughbreds, but also indicate the significant contribution of ROH segments dating further back before the formation of Thoroughbreds. Another recent study reported the estimates of ROH in a number of other horse breeds using the same 670k array with the same methods and settings to detect ROH segments [50]. In their study, the proportion of the genome covered by ROH segments ranged from 10.1% in Noriker and 17.7% for Purebred Arabian horses. These values are much lower than our estimate (24.5%) for Thoroughbreds. Higher inbreeding coefficients in Thoroughbreds compared to other breeds based on the observed versus expected number of homozygous genotypes has also been previously reported [28]. Interestingly, low correlation between *F*_*ROH*_ and *F*_*PED*_ was also observed for the other horse breeds (0.19 and 0.38 in Noriker and Austrian Haflinger horses, respectively) [50], suggesting that this might be a general feature in horses, possibly due to the severe bottleneck during the domestication of horses. Taken together, excessive inbreeding in domesticated horses before the establishment of many of the modern breeds may have contributed to the degree of inbreeding observed in the genomes of these breeds.

## Conclusions

Here, by performing population genetic analyses, we identified a number of regions that are likely to be associated with various important traits in Thoroughbreds, and showed that historical inbreeding has played an important role in shaping the genome of modern Thoroughbreds. Our results demonstrate the utility of horses as an interesting system to study genetics thanks to the presence of a high-density SNP array, detailed pedigree record, a large number of half-sibs, and several breeds with high phenotypic diversity. Future studies with a larger number of samples and detailed records of various phenotypic traits should enable us to understand the genetic basis of various phenotypic traits.

## Methods

### Samples, genotyping, and phasing

This research was approved by the Animal Care and Use Committee at Hidaka Training and Research Center. Blood samples were collected from 424 Thoroughbred racehorses owned by the Hidaka Training and Research Center (Japan Racing Association, JRA). All horses were born between 2009 and 2015. DNA was extracted by Maxwell 16 (Promega) with the Maxwell 16 LEV Blood DNA Kit. SNP genotyping was performed using the Axiom Equine Genotyping Array (Axiom MNEC670) which includes a total of 670,796 SNP markers. The data file containing all 670,796 SNP calls of the 370 samples are available as a VCF format file from EMBL-EBI (European Bioinformatics Institute, https://www.ebi.ac.uk, Project: PRJEB32686, Analyses: ERZ936110 at European Variation Archive). The genomic coordinates based on the equCab2 assembly is used throughout this article. 54 samples with an average genotype call rate of <98% were removed, resulting in 370 samples that were used in this study for genome-wide analyses. SNPs that could not be genotyped in >1% of the samples were removed, resulting in 536,448 SNPs that were used in this study. For phasing, *hsphase* [20, 21] was first run for each half-sib family with more than 10 half-sibs to impute the genotype of the sire and to phase the half-sibs. The phased genotypes of the half-sibs resulting from *hsphase* and the unphased genotypes of the remaining samples were combined into a single VCF file, and BEAGLE v4.1 [22] was run on this VCF file to phase the remaining samples.

### Population genetic analyses

Linkage disequilibrium (LD) was estimated by calculating *r*^2^ [23] between all SNPs within 1 Mb based on the phased haplotypes. The decay of LD against the physical distance was measured by calculating the average LD for each non-overlapping bins of 1 kb. Various population genetic analyses were performed using the SNPs from the 370 Thoroughbreds genotyped in this study and the SNPs from 311 samples containing various horse breeds from [19]. Out of the 670,796 SNPs, 496,245 SNPs that were genotyped in ≥99% of the samples in this study and the samples from [19] were retained for the following analyses. An unrooted Neighbor-Joining (NJ) tree [26] of the combined 681 samples was constructed based on a pairwise *p*-distance matrix with MEGA7 [51] and was visualized using iTOL v4 [52]. Bootstrap support for each branch was calculated based on 1,000 replicates. Principal component analysis (PCA) was performed by calculating the eigenvectors using the prcomp function of the R statistical package. The population structure was examined using STRUCTURE v2.3 [27] with a model assuming admixture, correlated allele frequency, and no linkage with *K* = 2, 8, 14 where *K* is the number of populations. For PCA and STRUCTURE, 10,607 SNPs with no missing data and an average distance between adjacent SNPs of ∼200 kb were used.

The heterozygosity was calculated for each site separately for the 370 Thoroughbreds of this study, 24 Thoroughbreds of [19], and the 287 non-Thoroughbred samples of [19]. The genome-wide distribution of conditional nucleotide diversity was obtained by calculating the average heterozygosity over an overlapping sliding window of 1 Mb with an increment of 200 kb. For the specific regions showing reduced diversity, the distribution was obtained by calculating the average diversity over an overlapping sliding window of 600 kb with an increment of 120 kb for the regions on chromosomes 1, 7, and 28, and over a window of 400 kb with an increment of 80 kb for the region containing *MC1R* on chromosome 3.

The population branch statistic (PBS) was calculated over an overlapping sliding window of 1 Mb with an increment of 200 kb (the same windows used to calculate the genome-wide distribution of the conditional nucleotide diversity) following [46] in order to detect changes in allele frequency specific to Japanese Thoroughbreds. Specifically, first, the *F*_*ST*_ values [53] were calculated for the Japanese Thoroughbreds from this study and the Thoroughbreds from Schaefer *et al*. [19], the Japanese Thoroughbreds and all the non-Thoroughbred horses from [19], and the Thoroughbreds of [19] and all the non-Thoroughbred horses. The *F*_*ST*_ values were then transformed to obtain estimates of the population divergence time *T* in units scaled by the population size following [54] as *T* = −*log*(1 − *F*_*ST*_). For each genomic window this value was calculated between the Japanese Thoroughbreds and the Thoroughbreds of Schaefer *et al*. (*T*^*JTST*^), the Japanese Thoroughbreds and the non-Thoroughbred horses (*T*^*JTnT*^), and the Thoroughbreds of Schaefer *et al*. and the non-Thoroughbred horses (*T*^*STnT*^). The length of the branch leading to the Japanese Thoroughbreds was then calculated as

$$\begin{array}{c} \begin{array}{c} PBS=\frac{T^{JTST}+T^{JTnT}-T^{STnT}}{2} \end{array} \end{array}$$ (1)

### Inbreeding coefficients

The pedigree-based inbreeding coefficients *F*_*PED*_ [55] for each individual were calculated based on the pedigrees of 5 and 10 generations. The pedigrees of all horses were obtained from www.netkeiba.com. Segments of runs of homozygosity (ROH) of >0.5 Mb and >5 Mb were detected using PLINK v1.07 [56] with the following settings: a minimum SNP density of one SNP per 50 kb, a minimum number of 80 homozygous SNPs, a maximum gap length of 100 kb, 1 heterozygote and 2 missing genotypes permitted, a minimum minor allele frequency of 0.01 for the SNP to be considered, and a minimum length of homozygous segment of 0.5 Mb or 5 Mb. The inbreeding coefficients *F*_*ROH*_ were calculated as the proportion of the genome covered by ROH segments assuming that the length of the genome is 2243 Gb.

### Genome-wide association studies

The coat color of all 370 horses were obtained from www.netkeiba.com. Association with the gray coat color was tested using the 18 gray horses as cases and the remaining 352 non-gray horses as controls. Association with the chestnut coat color was tested using the 94 chestnut horses as cases and the 258 non-gray, non-chestnut horses as controls. Association with racing performances was done by classifying high-performance horses and low-performance horses based on their lifetime earnings at JRA races. The racing records until the end of 2018 for all horses born before 2015 were obtained from www.netkeiba.com. Horses that had earned ≥30 million JPY were classified as high-performance horses and horses that had earned <5 million JPY despite having raced at least 3 times were classified as low-performance horses. In the JRA racing system, a win at a maiden race (a race between horses that have not won a race yet) earns ∼5 million yen. Horses that cannot win until the end of the age of 3 lose their eligibility to enter maiden races and often end up retiring. Thus, most if not all horses classified as low-performances will not be able to earn any more at JRA races. Genome-wide association studies were performed using GEMMA [39] by first estimating the centered genotype relatedness matrix and using a linear mixed model that incorporates this relatedness matrix. SNPs with MAF <0.05 were removed. The Wald test implemented in GEMMA was used to obtain the p-values.

## Supporting information

S1 Fig The decay of linkage disequilibrium. *r*^2^ is plotted as a function of distance in kilobase up to 1 Mb. The average *r*^2^ values over each non-overlapping 1 kb bin are shown.
(EPS)

S2 Fig Results of principal component analysis (PCA) controlling for the large sample size of Japanese Thoroughbreds. SNPs from a randomly selected subset of 10 Thoroughbreds genotyped in this study and the 311 samples from 20 breeds of [19] were used.
(EPS)

S3 Fig Neighbor-Joining tree with bootstrap values. SNPs from the 370 Thoroughbreds genotyped in this study and the 311 samples from 20 breeds of [19] were used. Bootstrap support based on 1,000 replicates is shown for major branches when greater than 70%. This tree is only for showing the bootstrap values. The tree is rooted at midpoint.
(TIF)

S4 Fig Results of STRUCTURE analysis of *K* = 2; 8; 14 controlling for the large sample size of Japanese Thoroughbreds. SNPs from a randomly selected subset of 10 Thoroughbreds genotyped in this study and the 311 samples from 20 breeds of [19] were used.
(TIF)

S5 Fig Haplotype block structures of the chromosomes inherited from the sire in a half-sib family inferred by *hsphase*. The two homologous chromosomes of the sire are shown at the top in red and blue, and the chromosomes inherited from the sire in each genotyped half-sib are shown below. Results of all chromosomes for each half-sib family with 10 or more half-sibs are shown.
(EPS)

S1 Table Search for targets of selection specific to Japanese Thoroughbreds. Protein-coding genes in the top 10 regions with the highest PBS values are listed.
(XLSX)
