# Supplementary figures and images for: Genome-wide SNP analysis of Japanese Thoroughbred racehorses
Source: PLoS One. 2019 Jul 24;14(7):e0218407. doi: 10.1371/journal.pone.0218407 (PMC6655603; doi:10.1371/journal.pone.0218407)

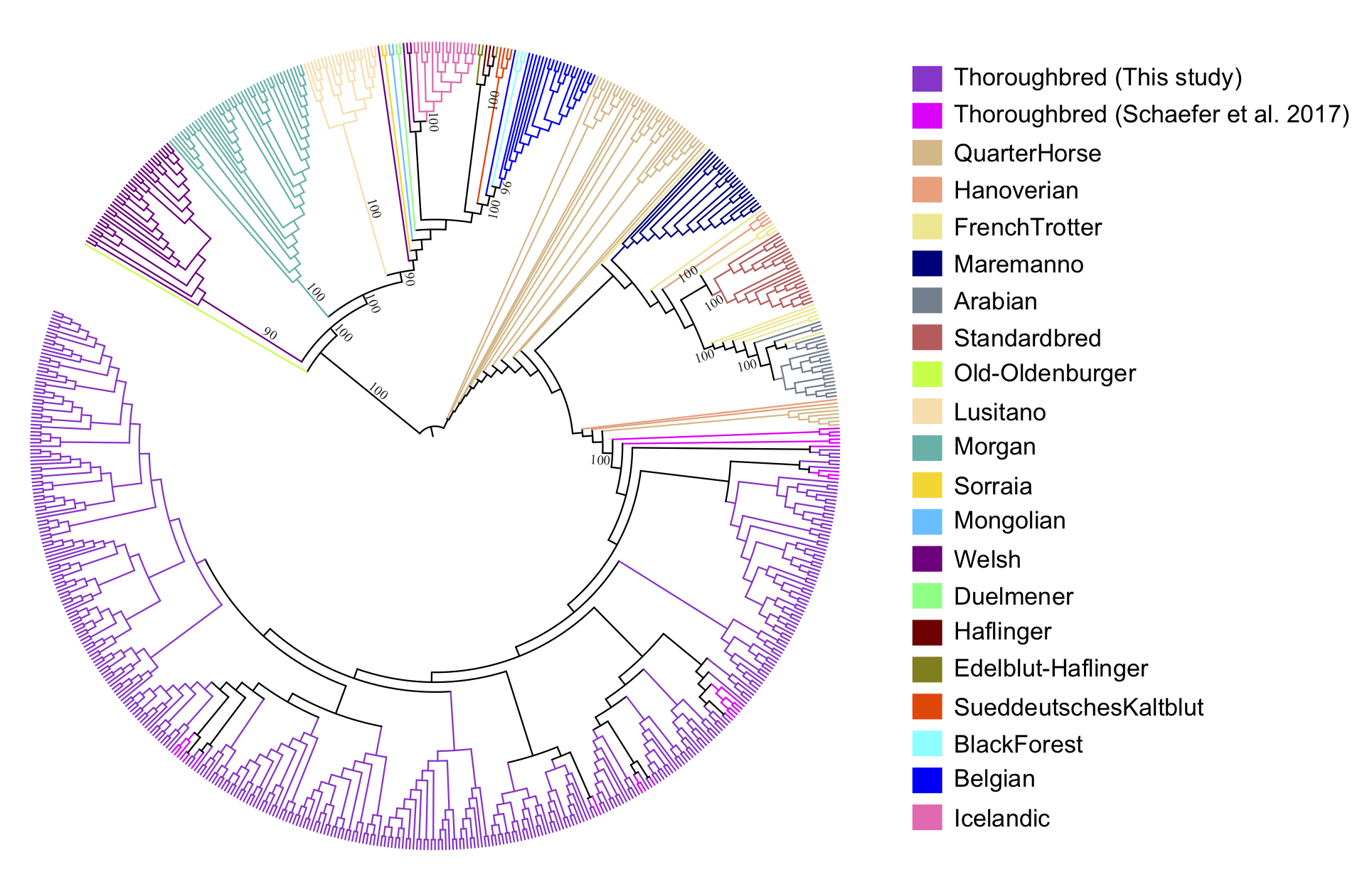

Supplement: S3 Fig — SNPs from the 370 Thoroughbreds genotyped in this study and the 311 samples from 20 breeds of [19] were used. Bootstrap support based on 1,000 replicates is shown for major branches when greater than 70%. This tree is only for showing the bootstrap values. The tree is rooted at midpoint. (TIF) [file pone.0218407.s003.tif]

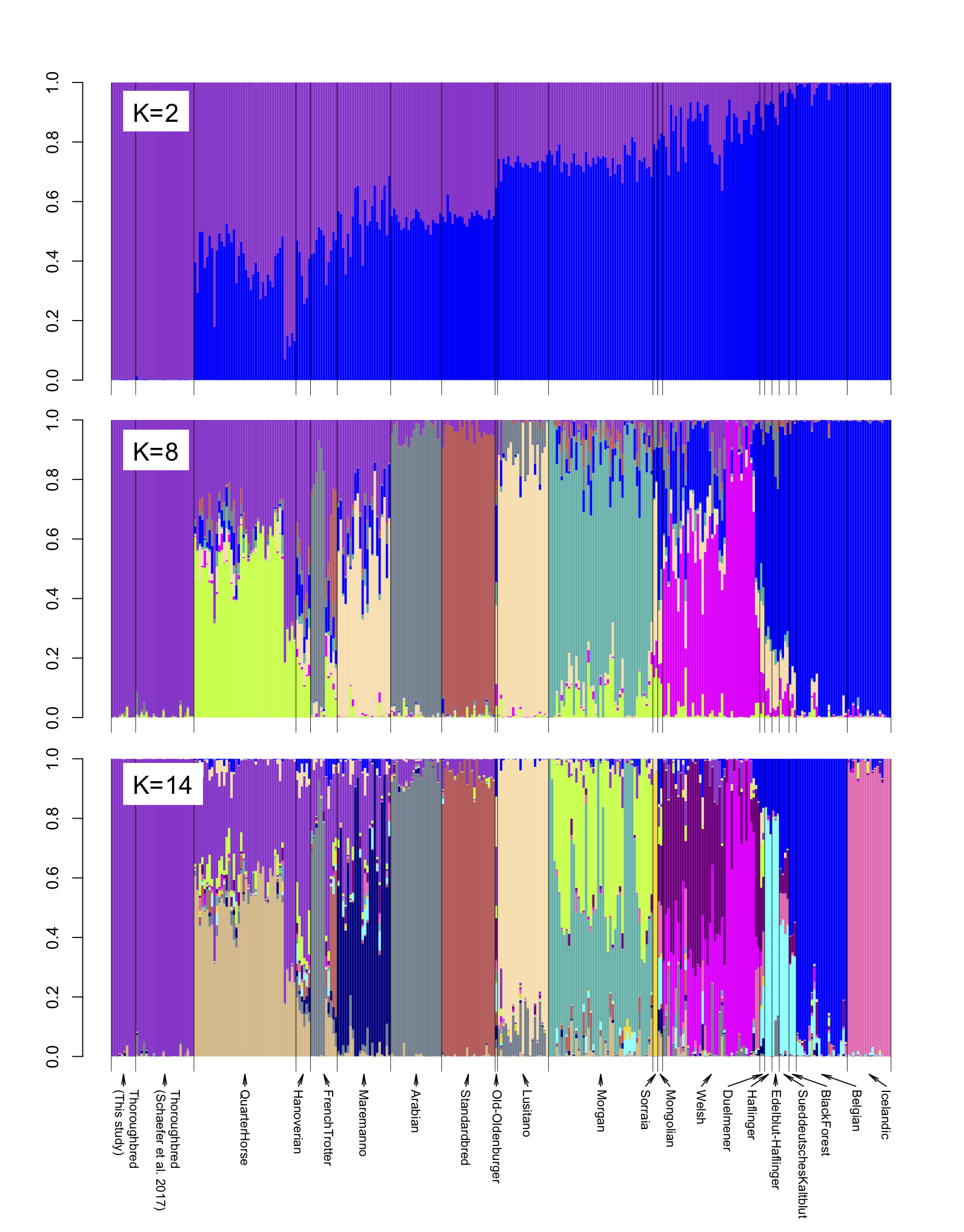

Supplement: S4 Fig — SNPs from a randomly selected subset of 10 Thoroughbreds genotyped in this study and the 311 samples from 20 breeds of [19] were used. (TIF) [file pone.0218407.s004.tif]
